# ONLINE MEDICAL RECORD (OMR) USE IN CAREGIVERS OF PEOPLE WITH DEMENTIA IN THE UNITED STATES

**DOI:** 10.1093/geroni/igad104.2838

**Published:** 2023-12-21

**Authors:** Megumi Inoue, Kyeung Mi Oh, Krista Beran, Emma Booker, Naoru Koizumi

**Affiliations:** George Mason University, Fairfax, Virginia, United States; George Mason University, Fairfax, Virginia, United States; George Mason University, Fairfax, Virginia, United States; George Mason University, Fairfax, Virginia, United States; George Mason University, Fairfax, Virginia, United States

## Abstract

**Objective:**

Studies have found that there are higher levels of stress among informal caregivers of individuals with dementia. As a result, it is important to seek ways to reduce their burden. Online Medical Records (OMRs), often referred to as patient portals, provide effective communication tools to caregivers and the use of OMRs have been found to have the potential to ease their stress levels. This study examined OMR usage in informal caregivers of people with dementia and identified factors that were associated with their usage.

**Methods:**

Data came from the HINTS, a National Cancer Institute (NCI)-sponsored survey of American adults. We merged data from HINTS 5 cycles 2, 3, and 4 administered between 2018 and 2020 into 1 data set. Our analytical sample included a total of 304 informal caregivers of people with Alzheimer’s disease. The outcome variable was OMR use in the last 12 months, and independent variables included age, sex, race and ethnicity, education, household income, marital status, employment status, relationship to care recipient, and the number of chronic conditions. Data was analyzed using multivariate logistic regression.

**Results:**

53% of caregivers have used OMRs at least once in the past 12 months. Caregivers with a higher level of education and greater number of chronic conditions are more likely to have used OMRs.

**Conclusion:**

Being able to access OMRs could reduce caregiver stress. There are certain caregiver characteristics that increase the likelihood of them utilizing this resource. Targeted approaches may be necessary to increase the range of use.

